# Changes in the Status of Spastic Diplegic Children in Terms of Gross Motor Function Classification System and Functional Mobility Scale Following Surgical Intervention: A Single Centre Experience

**DOI:** 10.7759/cureus.35105

**Published:** 2023-02-17

**Authors:** Laxmish R, Vikas Gupta, Nitu Mishra, Shubhangi Gupta, Prateek Behera

**Affiliations:** 1 Orthopaedics, Vardhman Mahavir Medical College and Safdarjung Hospital, New Delhi, IND; 2 Obstetrics and Gynecology, Gandhi Medical College, Bhopal, IND; 3 Obstetrics and Gynecology, Vardhman Mahavir Medical College and Safdarjung Hospital, New Delhi, IND; 4 Pediatrics, Vardhman Mahavir Medical College and Safdarjung Hospital, New Delhi, IND; 5 Orthopaedics, All India Institute of Medical Sciences, Bhopal, IND

**Keywords:** post operative rehab and soft tissues injuries, physical medicine and rehabilitation, treatment of cerebral palsy, cerebral palsy (cp), semls, fms, gmfcs, spastic diplegia

## Abstract

Introduction

Most centers in low- to mid-income countries (LMICs) lack facilities for a comprehensive instrumented gait analysis (IGA) which is often considered the preferred method for assessment of the functional results of surgery in children with spastic diplegia. We aimed to study if there were any changes in the Gross Motor Function Classification System (GMFCS) levels and Functional Mobility Scale (FMS) scores after surgery and whether they can be used as an indirect indicator of change in the functional status of a child.

Methods

This prospective study was conducted at the Pediatric Orthopedic unit of a teaching hospital on spastic diplegic children requiring surgical intervention. GMFCS levels and FMS scores were recorded before the surgery and at each follow-up visit, with the latest record being two years post-surgery. The change in the scores was indicated as an improvement, deterioration, or no change from the baseline and compared to the score of the preceding visit. In addition, it was examined whether the age at surgery had any effect on the temporal change in the scores.

Results

A total of 25 children were included for analysis after excluding those who failed to fulfill the predefined inclusion and exclusion criteria. Both the GMFCS levels and FMS scores improved from the third month to one-year post-surgery, after which a few patients had a worsening of their scores at the two years follow-up visit. The age at which surgery was performed had no significant effect on the pattern of change in the scores. Most children sought consultations with the physical therapy department only when they visited the surgical team for follow-up.

Conclusion

This study shows that surgical interventions do improve the functional outcomes in children with spastic CP when assessed using FMS scores while maintaining an undeteriorated GMFCS level in most children. While a peak improvement can be expected one year after surgery in most patients, possible of worsening from baseline scores do exist, and the parents must be informed of the same. Any decision for surgery must involve the parents, and the usefulness of postoperative physical therapy must be impressed upon them before the surgery and during each follow-up visit too.

## Introduction

Cerebral palsy (CP) is a common disorder of movement affecting children. Although it has a worldwide distribution, it is considered the most common condition responsible for physical disability in children in developed countries [1,2]. While the extent of the cerebral lesion (motor cortex involvement disrupting the transmission of signals) resulting in CP usually tends not to progress over time, the musculoskeletal manifestations often change. The temporal evolution of these musculoskeletal changes can affect a child’s physical abilities. Parents seek consultation from pediatricians, general physicians, physiatrists, and orthopedic surgeons for their child’s condition.

In the early years of life, management strategies are focused on improving the child’s ability to perform day-to-day activities while controlling abnormal movements [1]. Physiotherapy is the main treatment provided to achieve these goals. In the later years, improving the appearance and preventing pain also form a part of the management. Fixed deformities of the lower limb are often surgically addressed in a one-stage multilevel approach that can include lengthening of fixed muscle-tendon contractures, osteotomies, and correction of dynamic imbalance by tendon transfers. Selective dorsal rhizotomies have been reported to help address spasticity [1]. Additionally, surgical management aims not only to improve an individual’s gait and function but also to prevent possible deterioration [3]. Assessment of gait and function after any surgical intervention is necessary to evaluate the effectiveness of that intervention. Instrumented gait analysis (IGA), used in combination with clinical examination findings, is used for determining an appropriate surgical prescription. However, IGA is not widely available and often requires the support of trained therapists for interpretation [4]. Most clinicians caring for these children in low- to middle-income countries (LMIC) lack the facilities for IGA.

Gross Motor Function Classification System (GMFCS) is an instrument developed to classify the severity of functional limitation/disability in children with cerebral palsy [5]. Bodkin et al. [6] assessed the interrater reliability of GMFCS and reported that it has good interrater reliability and validity in children with CP. It is a five-level scale, and it rates a child’s gross motor function. It emphasizes movement initiation, sitting control, and walking for this purpose [5]. While level I represents the highest gross motor function, level V represents the lowest. The Functional Mobility Scale (FMS) was proposed by Graham et al. [7] based on the premise that an individual’s mobility can differ with a change in the environment. GMFCS is assessed by a clinician or a therapist in the hospital setting, and the FMS assessment is usually done by the parents/caregivers, who assesses the functional mobility of an individual in the community setting. While an IGA provides an objective and documentable assessment of gait changes, we believe that FMS can provide an indirect indication regarding the effectiveness of any procedure. The GMFCS level of a child usually tends to stay stable after surgery. Post-operative rehabilitation plays an important role in the functional improvement of these children [8,9].

The clinicians in LMICs often tend to rely on clinical judgment and observational gait analysis while offering surgical treatment. With this study, we aimed to examine if there was a change in the status of spastic diplegic children after surgery in their FMS scores and in the stability of their GMFCS level to help answer the question frequently asked by parents, i.e., whether their child would benefit from the surgery or not?

## Materials and methods

This prospective study was conducted after obtaining approval from the Institutional Ethics Committee in the Pediatric Orthopedics unit of the Orthopedics department at a tertiary care teaching hospital located in Northern India. Children operated on over two years between December 2016 and November 2018 were considered for enrollment. Children with spastic diplegic cerebral palsy between 4-18 years of age with GMFCS levels 2 and 3, who underwent single-event multilevel surgery (SEMLS) and who were capable of following commands, were included. Children with impaired mobility from other neuro-developmental causes, such as dystonia, chorea, or athetosis, progressive motor impairments, uncontrolled seizure disorders, and who were deemed unfit for anesthesia, were excluded. Additionally, children with GMFCS levels 1 and 4 and those who required subsequent additional surgical procedures or who failed to complete a minimum two-year follow-up were excluded too. The decision for surgical intervention was taken by the investigators after an adequate trial of non-operative management had been undertaken, and it was concluded that the non-operative means alone were inadequate for improving the functional capability any further. All the children with spastic diplegic CP were initially managed by physiotherapy and splints, as per the clinical judgment of the clinicians. While almost all the children were offered Baclofen tablets, Botulinum toxin injections were offered to selected patients depending on their suitability for the same. The non-operative treatment was continued for at least six months before offering surgery. Consent for participation was obtained from the parents or legal guardians before enrolling the children in the study.

All the surgeries were performed by the same surgeon, with the quantum of the surgery decided based on the physical examination and observational gait evaluation. After the surgery, cast immobilization was employed for usually six weeks. Appropriate orthosis was prescribed after the cast removal. Physical exercises were decided individually and started after the cast removal by the physical therapists. Ambulation was started after the application of an orthosis and with the help of walking aids. Parents were advised to continue exercises and walking at home and were called for follow-up at regular intervals with both the surgical team and the physical therapists.

One of the investigators who was not involved in the surgeries assessed the GMFCS and FMS scores during the follow-up visits. The GMFCS levels and FMS scores were obtained in the pre-operative period (baseline values) and at three months, six months, one year, and two years after the surgical intervention. The values at each follow-up visit were interpreted in terms of improvement, deterioration, or no change for both scores. For evaluating whether the age at surgery influenced the outcomes, the patients were divided into two categories, namely less than 12 years old (<12 years old); and more than or equal to 12 years old (>= 12 years old). The data was collected in a predefined proforma on a Microsoft Excel (Microsoft corp., Redmond, WA) spreadsheet. Data analysis and preparation of tables and graphs were then done using Microsoft Excel and IBM Corp. Released 2019. IBM SPSS Statistics for Windows, Version 26.0. Armonk, NY: IBM Corp. The results have been presented in terms of numbers (with % in parentheses), frequencies for GMFCS and FMS scores, and range, mean, and standard deviation for age. Independent samples Mann-Whitney U test was applied to analyze if the gender of the patients or their age at surgery had any effect on the GMFCS and the FMS scores. The statistical significance was set at p<0.05.

## Results

Thirty-two patients satisfying the inclusion criteria were operated upon during the study period. However, after excluding those who failed to satisfy the inclusion and exclusion criteria, 25 patients were included for analysis. There were 16 males and nine females (Table 1).

**Table 1:** Table depicting the age, gender characteristics, and GMFCS levels of the patients. The values mentioned in the parentheses are the percentage of the total of each category. GMFCS: Gross Motor Function Classification System

Particulars		Numbers (% in parentheses)
Gender	Male	16 (64) – 8 (aged less than 12 years) and 8 (aged more than or equal to 12 years)
	Female	9 (36) – 4 (aged less than 12 years) and 5 (aged more than or equal to 12 years)
Age Distribution	Less than 12 years	12 (48)
	More than or equal to 12 years	13 (52)
Baseline GMFCS (Level)	3	13 (52)
	2	12 (48)
GMFCS at 3 months (Level)	4	1 (4)
	3	6 (24)
	2	15 (60)
	1	3 (12)
GMFCS at 6 months (Level)	3	6 (24)
	2	13 (52)
	1	6 (24)
GMFCS at 1 year (Level)	3	3 (12)
	2	15 (60)
	1	7 (28)
GMFCS at 2 years (Level)	4	1 (4)
	3	5 (20)
	2	15 (60)
	1	4 (16)

The mean age of the patients was 10.96 years (4-18 years). The surgical dose varied with each patient. The most frequently performed procedures were hip adductor release (n=20), fractional lengthening of the medial hamstrings, and lengthening of the gastrocnemius-soleus (Strayer’s/Vulpius/Baker’s procedure). Three patients underwent fractional lengthening of the Iliopsoas in addition to the procedure on the hamstrings and calf muscles. Two patients had lengthening of the rectus femoris, while one had distal femoral derotational osteotomy in addition to other procedures.

Table 1 depicted above shows the number of patients with different GMFCS levels before the surgery (baseline) at three months, six months, one year, and two years. Table 2 presents in number (with %) of patients with different FMS scores during each follow-up. The temporal change in the FMS (5m, 50m, and 500m) scores has also been presented in this table.

**Table 2:** Details of the grades assigned to the 5m, 50m, and 500 m components of the FMS during different follow-up visits of the patients. FMS: Functional Mobility Scale

Time	FMS grades	FMS, N (%)		
		5m	50m	500m
Baseline	5	15 (60%)	11 (44%)	10 (40%)
	4	9 (36%)	11 (44%)	11 (44%)
	3	-	3 (12%)	3 (12%)
	2	1 (4%)	-	1 (4%)
3 months	6	3 (12%)	3 (12%)	3 (12%)
	5	13 (52%)	11 (44%)	11 (44%)
	4	7 (28%)	7 (28%)	7 (28%)
	3	1 (4%)	2 (8%)	2 (8%)
	2	2 (4%)	2 (8%)	2 (8%)
6 months	6	6 (24%)	6 (24%)	6 (24%)
	5	12 (48%)	10 (40%)	9 (36%)
	4	7 (28%)	6 (24%)	7 (28%)
	3	-	2 (8%)	2 (8%)
	2	-	1 (4%)	1 (4%)
1 year	6	7 (28%)	7 (28%)	7 (28%)
	5	15 (60%)	10 (40%)	8 (32%)
	4	3 (12%)	8 (32%)	9 (36%)
	3	-	-	1 (4%)
2 years	6	4 (16%)	4 (16%)	4 (16%)
	5	14 (52%)	11 (44%)	11 (44%)
	4	6 (24%)	7 (28%)	7 (28%)
	3	-	1 (4%)	1 (4%)
	2	1 (4%)	2 (8%)	2 (8%)

An analysis of the pattern of change in the GMFCS levels shows that at three months post-surgery, nine patients (36%) had improvement in GMFCS by one level from the baseline value; one patient had deterioration of one level, and 15 (60%) had no improvement. Further details of the number of patients having improvement, deterioration, or no change from the previous and the baseline values during each follow-up are presented in Table 3. Based on this data, one can note that the maximum improvement was achieved one year after the surgical interventions and that no patient had any further improvement after that. At two years post-surgery, six (24%) patients had deterioration of their GMFCS level from the score recorded one year ago. Overall, after two years of surgical intervention, 11 (44%) had an improvement from their baseline level, one had deterioration, and 13 (52%) had no change.

**Table 3:** Summary of the number (N) of patients with changes (improvement, no change or deterioration) in their GMFCS levels and FMS scores during the study period. ‘p’ values mentioned are of Wilcoxon two related samples non=parametric test GMFCS: Gross Motor Function Classification System; FMS: Functional Mobility Scale; ‘*’ and ‘#’ are based on the improvement and deterioration of GMFCS levels, respectively. ‘^’ and ‘++’ are based on the improvement and deterioration of FMS scores, respectively.

		GMFCS		FMS N (%)					
		N (%)	‘p’ value	5m		50m		500m	
				N (%)	’p’ value	N (%)	‘p’ value	N (%)	‘p’ value
3 months	Improvement from baseline score	9 (36%)	0.011^*^	7 (28%)	0.359^^^	7 (28%)	0.492^^^	9 (36%)	0.226^^^
	No change from baseline score	15 (60%)		15 (60%)		16 (64%)		14 (56%)	
	Deterioration from baseline score	1 (4%)		3 (12%)		2 (8%)		2 (8%)	
6 months	Improvement from baseline score	12 (48%)	0.001^*^	10 (40%)	0.008^^^	11 (44%)	0.021^^^	12 (48%)	0.013^^^
	No change from baseline score	13 (52%)		14 (56%)		13 (52%)		12 (48%)	
	Deterioration from baseline score	0		1 (4%)		1 (4%)		1 (4%)	
	Improvement from score at 3 months	4 (16%)	0.059^*^	6 (24%)	0.020^^^	5 (20%)	0.034^^^	4 (16%)	0.059^^^
	No change from score at 3 months	21 (84%)		19 (76%)		20 (80%)		21 (84%)	
	Deterioration from score at 3 months	0		0		0		0	
1 year	Improvement from baseline score	16 (64%)	0.000^*^	14 (56%)	0.000^^^	15 (60%)	0.000^^^	14 (56%)	0.000^^^
	No change from baseline score	9 (36%)		11 (44%)		10 (40%)		11 (44%)	
	Deterioration from baseline score	0		0		0		0	
	Improvement from score at 6 months	4 (16%)	0.046^*^	5 (20%)	0.025^^^	5 (20%)	0.034^^^	3 (12%)	0.102^^^
	No change from score at 6 months	21 (84%)		20 (80%)		20 (80%)		22 (88%)	
	Deterioration from score at 6 months	0		0		0		0	
2 years	Improvement from baseline score	11 (44%)	0.004^*^	10 (40%)	0.115^^^	8 (32%)	0.109^^^	11 (44%)	0.042^^^
	No change from baseline score	13 (52%)		12 (48%)		15 (60%)		12 (48%)	
	Deterioration from baseline score	1 (4%)		3 (12%)		2 (8%)		2 (8%)	
	Improvement from score at 1 year	0	0.020^#^	0	0.011^++^	0	0.008^++^	1 (4%)	0.035^++^
	No change from score at 1 year	19 (76%)		18 (72%)		17 (68%)		17 (68%)	
	Deterioration from score at 1 year	6 (24%)		7 (28%)		8 (32%)		7 (28%)	

The 5m, 50m, and 500m components of the FMS followed an almost similar pattern in terms of improvement or deterioration from the baseline scores. While the scores in the patients started to improve from the first re-assessment itself, i.e., at three months after the surgery, the improvement attained at one year of surgery could not be sustained in most patients at the two-year mark. At one year follow-up, almost 56% of the patients had improvement in all three components (5m, 50 m, and 500 m) of the FMS scores from the baseline values; there was no change in these scores in almost 44% of patients. These details are presented in Table 3. At two years of follow-up, 32% of patients had improvement in all three components of the FMS from the baseline value. But 8% of patients were noted to have deterioration from the baseline values.

Thus, the temporal pattern of change in the GMFCS and FMS scores was usually of an improvement during the follow-up, which was most pronounced at one-year follow-up. A few patients also had worsening of their scores at the final follow-up when compared to their baseline scores. There was no statistically significant difference in the pattern of change in the GMFCS and FMS scores in relation to age (Table 4) at which a patient underwent the surgical procedure (p values >0.05 on independent samples Mann Whitney U test).

**Table 4:** Several patients with a change from the baseline in GMFCS and FMS scores over time in relation to their age at surgery. GMFCS: Gross Motor Function Classification System; FMS: Functional Mobility Scale.

Follow - up		GMFCS		FMS					
				5m		50m		500m	
		<12 years	>= 12 years	<12 years	>=12 years	<12 years	>=12 years	<12 years	>=12 years
3 months	Improvement	4	5	2	4	3	4	4	5
	No change	8	7	8	7	9	7	8	6
	Deterioration	-	1	2	2	-	2	-	2
6 months	Improvement	5	7	4	6	3	7	5	7
	No change	7	6	7	7	9	5	7	5
	Deterioration	-	-	1	-	-	1	-	1
1 year	Improvement	7	9	6	8	6	9	6	8
	No change	5	4	6	5	6	4	6	5
	Deterioration	-	-	-	-	-	-	-	-
2 years	Improvement	4	7	3	7	3	6	4	7
	No change	7	6	7	5	8	6	7	5
	Deterioration	1	-	2	1	1	1	1	1

## Discussion

Švehlík et al. [4] stated that the interaction of joint contractures, muscle weakness, bony deformities, and joint instability at multiple levels can affect the quality and efficiency of gait in children with CP, which can then affect the child’s function in society as an individual. While Orthopedic surgeons have always played a role in offering surgical services for improving gait in children with CP, there has been a change in the way these surgeries are performed. The cycle of step-by-step correction of deformities followed by periods of rehabilitation, which has been termed the "Birthday syndrome" in literature [4], has paved the way for SEMLS. SEMLS aims to target all the addressable deformities in one surgical session and consists of soft-tissue and/or bone procedures (either alone or in combination). To be labeled SEMLS, the surgical team must have performed at least two procedures at two or more surgical sites in one anesthetic sitting in a child with CP [4,8,9]. SEMLS is the widely accepted method of managing children with spastic diplegia and has been shown to improve function in previous studies [9-14]. Most published studies have also mentioned the important role of post-operative rehabilitation, which should be the ideal scenario after any surgery [9, 12-14]. In the present study, a greater number of male patients were operated on than females and an almost equal number of patients of ages <12 years and >=12 years were operated on.

All the children, after removal of the casts, were on a home-based exercise regime that involved the exercises being performed by the parents or caregivers as per the instructions of physical therapists and fine-tuning of the exercises based on the evaluation during the follow-up visits with them. An important observation was that while all 25 patients followed up with the surgical team for the prescribed durations, most of them (21 out of 25) followed up with the physical therapy department only during their visits to the Orthopedic team and missed out on the other in-between visits required for rehabilitation. The parents were contacted telephonically for the follow-up visits, but still, the visits were not as frequent as recommended. The reasons provided by the parents for the non-compliance included financial constraints, a lack of time off from work, caring for the siblings of the patient, the non-availability of a social support system, and the relatively long distance of their residence from the hospital. These are often the common reasons for incomplete follow-up in other surgical and medical disciplines, too [14-16]. With this background, we presume that most patients probably didn’t receive adequate supervised rehabilitation and that it would be difficult to compare the results of this study with other studies in which children have received adequate rehabilitation.

The GMFCS level changes noted during this study followed the pattern of gradual improvement till one year, followed by no further change from one to two years in most patients. One year after the surgery, 62.9% of patients had an improvement from the baseline score, with 37% showing no change. At two years, however, seven (26%) and one patient had deterioration of GMFCS levels from the level at one year and the baseline, respectively. Overall, 11 (40,7%) patients had improvement in GMFCS from baseline. Four, two, and two patients had worsening of their FMS scores at three months, respectively, for the 5m, 50, and 500m components. The pattern of the temporal evolution of the FMS scores was, however, like that of GMFCS scores.

We thus noted an improvement in the GMFCS and FMS scores after surgical procedures. This improvement is consistent with the findings of other studies that have evaluated the role of SEMLS in bringing about a change in gait and function [4,6,12-14,17,18]. Gorton et al. [19], in their multicentric prospective comparative study, evaluated the gait and function of patients with CP who had undergone surgery with matched patients who were not operated upon. They concluded that there were significant improvements in gait kinematics from baseline to follow-up (at one year) for the surgical group compared with the nonsurgical group. Though the present study does not employ a similar comparison, we have shown that the FMS and GMFCS scores do improve the most one year following surgery. However, we failed to notice any effect of age at the surgery on the FMS and GMFCS scores. This is in contradiction to the results reported by Švehlík et al. [4], who concluded that children between 10-12 years were the benchmark responders to SEMLS in the long term in their study. While an exact reason for this variation in our study cannot be elucidated, the inadequacy of physical therapy might influence the outcomes.

While the results have been simply presented in terms of change in the FMS and GMFCS scores over time for ease of understanding by most clinicians, there are a few important limitations to this study. The number of patients included is small; thus, the results cannot be generalized to all children with spastic diplegic CP. No details of the initial and post-surgical muscle strength levels were collected. Detailed study instruments for objective assessment used in previously reported studies were not used. Detailed statistical analysis and mathematical modeling, as has been commonly done in other studies, were not performed. We also acknowledge that the follow-up duration was short as a long-term follow-up, as presented by Thomason et al. [3] and Švehlík et al. [4], would have been better. As probably most patients didn’t receive adequate rehabilitation, and muscular procedures without physical therapy might be inadequate, the generalization of the results needs to be performed with caution. However, many surgeons in LMIC might have faced a similar issue in their practice, and we believe that they would benefit from the results of this study. This study needs to be expanded further by involving other institutes with children of different socio-economic and cultural backgrounds included to critically appraise the results of this study and address its limitations.

## Conclusions

Despite its limitations, this study has shown that in the short term, surgical procedures in children with spastic diplegic CP tend to improve their function while maintaining the GMFCS at a reasonably stable level. We additionally attempted to answer the question that many Orthopedic surgeons dealing with children with CP in LMIC might have asked themselves while caring for patients whether they should offer surgery to the patient or not. Most of them probably do not have the resources and support system to ensure that the child receives adequate post-operative rehabilitation. Based on the results of this study, we believe that the parents and caregivers can be presented with a realistic picture of a possible improvement in functional status in the short term with the possibility of deterioration, and a final decision on surgery must be taken after due discussion with the parents. A further expansion of the study with a similar methodology but with even better compliance with physical therapy is necessary to evaluate the importance of physical therapy.
